# Oxidative Stability and Genotoxic Activity of Vegetable Oils Subjected to Accelerated Oxidation and Cooking Conditions

**DOI:** 10.3390/foods12112186

**Published:** 2023-05-29

**Authors:** Diana Ansorena, Rubén Ramírez, Adela Lopez de Cerain, Amaya Azqueta, Iciar Astiasaran

**Affiliations:** 1 Department of Nutrition, Food Science and Physiology, Center for Nutrition Research, Faculty of Pharmacy and Nutrition, University of Navarra, Irunlarrea 1, 31008 Pamplona, Spain; 2 IdiSNA, Navarra Institute for Health Research, Irunlarrea 3, 31008 Pamplona, Spain; acerain@unav.es (A.L.d.C.); amazqueta@unav.es (A.A.); 3 Department of Pharmacology and Toxicology, Faculty of Pharmacy and Nutrition, University of Navarra, Irunlarrea 1, 31008 Pamplona, Spain

**Keywords:** vegetable oils, fatty acids, oxidation, MiniAmes, mutagenicity

## Abstract

The oxidative stability and genotoxicity of coconut, rapeseed and grape seed oils were evaluated. Samples were submitted to different treatments: 10 days at 65 °C, 20 days at 65 °C (accelerated storage) and 90 min at 180 °C. Peroxide values and thiobarbituric acid reactive substances values were altered as a function of storage time, but their greatest changes were recorded in samples subjected to 180 °C. Fatty acid profiles did not show significant changes from the nutritional point of view. Volatile compounds showed the highest increases at 180 °C for 90 min (18, 30 and 35 fold the amount in unheated samples in rapeseed, grape seed and coconut oils, respectively), particularly due to the increment in aldehydes. This family accounted for 60, 82 and 90% of the total area in coconut, rapeseed and grapeseed oil, respectively, with cooking. Mutagenicity was not detected in any case in a miniaturized version of the Ames test using TA97a and TA98 *Salmonella typhimurium* strains. Despite the increment in the presence of lipid oxidation compounds in the three oils, they were not compromised from the safety perspective.

## 1. Introduction

Concerns about health and sustainability aspects have given rise to research dealing with vegetable-origin food as an alternative to animal-origin food. Vegetable oils have been studied during the last decades, with great emphasis on their healthy aspects due to their high amounts of unsaturated fatty acids and also of interesting unsaponifiable compounds including tocopherols, carotenoids, hydrocarbons or sterols. The dietary guidelines of the American Heart Association recommend the use of liquid plant oils to improve cardiovascular health [1].

Oxidation is one of the main chemical deterioration processes occurring during the self-life of foods. It is a key process to be controlled in oils to guarantee their nutritional value and safety. In this sense, the importance of examining the oxidative stability of commercially available edible oils through the analysis of the oxidation products formed during the simulated shelf life of different oils from animal, algae and vegetable origin has been pointed out [2]. However, proper measurement of lipid oxidation remains a challenging task, since the process is complex and depends on the type of lipid substrate, the oxidation agents and the environmental factors, among others [3]. It is known that oils or fats rich in unsaturated fatty acids are more prone to suffer oxidation than those with high levels of saturated fatty acids. Lipid oxidation can take place when high temperatures are applied, but also at room temperature, giving rise to a broad variety of compounds, including volatile compounds [4]. The toxicity of some of these volatile compounds, especially aldehydes, has been studied, and some of them have been considered possible causal agents of diseases such as atherosclerosis and cancer [5].

The different compositions of oils, both in the saponifiable and unsaponifiable fractions determine the intensity of oxidation and the type of compounds formed during their storage and culinary treatment. In particular, different fatty acid profiles have been found to induce different oxidation patterns [6]. Rapeseed oil (obtained from *Brassica campestris* seeds) is the second most abundant edible oil produced worldwide, after soybean oil [7], being especially rich in MUFA (65% oleic acid), followed by linoleic acid. Although it has traditionally been an oil with a high amount of erucic acid, new breeding methods have made it possible nowadays to obtain new varieties with low amounts of this fatty acid, a fact that has significantly contributed to the increase in its production and its economic worldwide impact [8]. Moreover, it has been suggested as an acceptable alternative to olive oil in areas with climate and agricultural background that support its production [9] Coconut oil (obtained from *Cocos nucifera*) is rich in medium-chain fatty acids, including caproic, caprylic, and capric acids and mainly lauric acid, being a highly saturated fat (90%). It is considered a good digestibility oil [10] and it is widely used as food in the world [11]. This oil was used in repeated frying of potato chips and was found to deteriorate less than soybean and olive oil, pointing out that it might be a better frying oil than the other oils for repeated use [12]. Grape seed oil (obtained from *Vitis vinifera* seeds) is a byproduct of the wine-making industry that has acquired an interest recently because of its potential health benefits [13,14,15]. It is highly unsaturated, with PUFAs reaching approximately 90% of the total fat content; linoleic acid is the most abundant fatty acid [16]. It could be expected that the different unsaturation degrees of these different oils could give rise to different behavior and oxidation patterns during their storage and heating treatments. The literature describes the modification of oxidation compounds by the application of high-temperature conditions [6,12,17,18] or in long-term low-temperature experiments [19,20,21,22,23]. However, no previous studies have been found to report a combination of different oxidation evaluation methods in oils with such different profiles as those selected in this work. Neither on the comparison of their behavior during accelerated shelf-life tests and high-temperature conditions.

Frying is a culinary technique commonly used in the Mediterranean gastronomic culture. When fats and oils are used for a deep-fat frying process, thermal and oxidative degradation occur, which can result in the formation of volatile and non-volatile degradation products [24]. Heating treatments have been shown to completely change the volatile organic compounds content of commonly used edible oils (extra virgin olive oil, pomace oil, soy oil, palm oil) [25], and other less common oils such as safflower and coconut [26]. In addition, the assessment of the long-term treatment of oils at high temperatures has also contributed to elucidating volatile markers of oxidation [6]. The continuous heating of edible oils is associated with adverse health effects. Cooking techniques such as frying, have been associated with an increased risk of carcinogenicity and genotoxicity [27,28]. Moreover, in extensive work dealing with the potential adverse health effects related to toxic compounds derived from lipid oxidation during frying, the urgent need for nutritional and epidemiological trials probing the relationships between the incidence of NCDs (Non-Communicable Diseases), and the frequency and estimated quantities of dietary intake of those compounds has been highlighted [29].

To assess the genotoxicity of undesirable substances in food products, two tests are recommended, as they identify different types of mutations: a bacterial reverse mutation assay and an in vitro micronucleus test [30]. The *Salmonella typhimurium* reversion test, commonly known as the Ames test, is the most widely used to detect gene mutations [31]. According to OECD guideline 471, a battery of five different tester strains is proposed for a complete assay, but a reduced number of bacterial strains can also be used for screening purposes or preliminary information [32]. The scientific evidence regarding the potential genotoxicity of vegetable oils under different conditions is rather limited. Positive results have been obtained in the Ames test with the edible palm oil after heating [33], but negative results were obtained with sunflower oil [34] or aqueous extracts of various vegetable oils [35].

The aim of the study was to verify the stability of the saponifiable fraction of three vegetable oils with different fatty acid profiles (coconut, rapeseed and grape seed) subjected to accelerated oxidation and cooking monitoring conditions, and to assess with a simple test their potential mutagenicity.

## 2. Materials and Methods

### 2.1. Materials

The three oils used in this study were coconut oil (Organic extra virgin coconut oil from PLANTIS^®^, Barcelona, Spain), rapeseed oil (Organic cold pressed rapeseed oil Terpenic lab S.L., Barcelona, Spain) and grape seed oil (Refined grape seed Terpenic lab S.L., Barcelona, Spain). Three different batches of each type of oil were purchased in a local market.

2-thiobarbituric acid, and fatty acid methyl esters were obtained from Sigma-Aldrich Chemical (Steinheim, Germany). Boron/methanol trifluoride and heptane were purchased from Merck (Whitehouse Station, NJ, USA). Potassium hydroxide, hexane, cyclohexanone, hydrochloric acid, trichloroacetic acid and ammonium sulfate were from Panreac (Barcelona, Spain).

*Salmonella typhimurium* strains TA97a and TA98 and the Mutazime S9 mix at 10% from livers of Aroclor 1254-induced rats were purchased from Moltox (Boone, NC, USA). Nutrient broth and Phosphate Buffered Saline (PBS) tablets were obtained from Oxoid (Basingtone, UK). 4-nitro-o-phenylenediamine (NPD), 2-aminoanthracene (AA) and 2-aminofluorene (AF), histidine and biotin were obtained from Sigma-Aldrich.

### 2.2. Accelerated Storage and Cooking Conditions

Oils were analyzed in crude conditions (T0), and after being subjected to the next treatments: 65 °C for 10 days (T1), 65 °C for 20 days (T2) (both were Schaal oven conditions) and 180 °C during 90 min (T3) (mimicking reiterative use of oils for frying foods). Treatments were carried out as follows: 2 g of oil was weighed into 25 mL glass vials. For Schaal oven conditions: the vials were placed in an oven at 65 °C, and sampling was conducted after 10 and 20 days. In these accelerated oxidation conditions (65 °C), one day is equivalent to one month at room temperature [20]. For cooking, the vials were placed in an orbital shaker (JP Selecta S.A., Rotaterm, Barcelona, Spain) at 180 °C for 90 min. Once the treatments were completed, the vials were sealed (the air was replaced with nitrogen to control the lipid oxidation process) and immediately cooled to room temperature and then stored in the freezer (−20 °C) until analysis, except for the volatile compound analysis, which was performed immediately after the heating treatments.

From each type of oil, three batches were submitted to each treatment (unheated oil, accelerated storage and cooking). Each parameter was analyzed in triplicate in the 12 different samples available per oil, using individual vials for every replicate.

### 2.3. Peroxides and TBARs

The peroxide value (PV) and TBARs were analyzed in oils spectrophotometrically. Briefly, for PV, 10 mg of oil was transferred to a tube and solved in a mixture of butanol/methanol (2:1). Ammonium thiocyanate (25 µL at 30%) was added and mixed. Then, 25 µL of ferrous chloride (36 mM in HCl) was added. Absorbance was measured at 510 nm after 15 min in the dark. In the case of TBARs, oil (0.25 g) was added with 0.4 mL of distilled water, 1 mL of thiobarbituric acid and 10 µL of butylhydroxytoluene. After the reaction (boiling for 15 min) and cooling, colored complexes were extracted with a mixture of 2 mL of cyclohexanone and 0.5 mL of ammonium sulfate. After centrifugation, absorbance was read at 532 nm. Both methods are described in detail elsewhere [20].

### 2.4. Fatty Acid Profile

Fatty acids (FAs) were determined in the assayed oils by gas chromatography FID detection [36], previous preparation of the fatty acid methyl ester derivatives using boron trifluoride/methanol. A Perkin–Elmer Clarus 500 gas chromatograph (Shelton, CT, USA), equipped with a split–splitless injector, and automatic autosampler, and coupled to a computerized system for data acquisition (TotalChrom, version 6.2.1) was used. It was fitted with a capillary column SPTM-2560 (100 m × 0.25 mm I.D. × 0.2 μm). The temperature of the injection port was 250 °C and 260 °C for the detector. The oven temperature was programmed to increase from 170 to 200 °C at a rate of 10.0 °C/min and then at a rate of 4.0 °C/min to 220 °C. The carrier gas was hydrogen, 2.15 mL/min. The sample size was 0.5 μL and the split ratio was 120:1. The identification of the 30 fatty acids analyzed was conducted by the comparison of their retention times with those of pure fatty acid methyl esters. The quantification used heptadecanoic acid methyl ester as an internal standard. Individual methylated standards from Sigma (St. Louis, MO, USA) were used for the saturated, monounsaturated, cis polyunsaturated fatty acids and the trans-t-Palmitoleic C16:1 Δ9t, Elaidic C18:1 Δ9t, Brassidic C20:1 Δ13t. For Linoleic acid isomers, the mixture of Linoleic acid cis/trans isomers (50% of C18:2Δ9t,12t; 20% of C18:2Δ9c,12t and C18:2Δ9t,12c; 10% of C18:2Δ9c,12c) from Sigma was used. The order of elution in the case of mixtures of isomers (Linoleic acid cis/trans isomers) was also considered [37], and spiking the sample with each standard individually was finally used for confirming the identification. Elaidic acid eluted very closely to other C18:1 trans isomers (possibly Δ6–Δ12), which are all located before Oleic acid (C18:1 Δ9c). Quantification for all these C18:1 trans isomers was conducted as the sum of all of them. After the quantification of the individual FA, the sums of saturated (SFA), monounsaturated (MUFA), polyunsaturated (PUFA), trans, ω-3 and ω-6 were also calculated.

### 2.5. Volatile Compounds

Volatile compounds were analyzed by headspace solid phase microextraction (HS-SPME) combined with gas chromatography–mass spectrometry (GC–MS) as described in Tura et al. [20] and Gutiérrez-Luna et al. [38]. The SPME fiber coating used was divinylbenzene/carboxen/polydimethylsiloxane (DVB/CAR/PDMS) (50/30 µm film thickness, Supelco). Vegetable oil (2 g) was weighed in a 25 mL headspace vial. The sample was equilibrated at 50 °C for 15 min and the adsorption time, with the fiber exposed to the headspace of the sample, was 60 min at the same temperature. The desorption time for the fiber in the injection port of the gas chromatograph was 30 min. The GC–MS instrumentation used was GC 6890 N coupled to a mass selective 5973 detector (Agilent Technologies, Santa Clara, CA, USA). Volatiles were separated using a capillary column HP-5MS, 5% phenylmethylsiloxane (30 m × 0.25 mm I.D. × 0.25 µm, from Agilent Technologies, Santa Clara, CA, USA). Chromatographic conditions were as follows: the oven temperature was held for 5 min at 42 °C, then increased to 120 °C at 3 °C min^−1^ and to 250 °C at 10 °C min^−1^ (5 min hold); injector temperature, 250 °C; detector temperature 280 °C; ion source temperature, 230 °C; quadrupole mass analyzer temperature, 150 °C. Helium was used as carrier gas at 1 mL min^−1^. The mass spectrometer was operated by electronic impact at 70 eV and ions were scanned over the *m*/*z* range of 33–350 at a rate of 4.43 scan/s.

The identification of each peak was made taking into account the KI (Kovats Index) reported in the literature, and comparing their mass spectra with the one of a commercial library (Wiley^®^, Mass Spectral Database). A semi-quantitative approach was used for quantification, measuring TIC (Total Ion Chromatogram) area and, in the case of overlapping peaks, the quantification of the corresponding compound was conducted by integrating the area of the specific ion and considering the relative ratio in which this ion is present in each compound. Results are expressed in area × 10^3^/g oil. Samples were analyzed in triplicate.

### 2.6. Mutagenicity Test

The Ames test was carried out as previously described by Maron and Ames [39], but it was adapted to a miniaturized version in 6-well plates to reduce the test product needed. The assay based on Burke et al. [40] with some modifications, was performed with *Salmonella typhimurium* strains TA97a and TA98 at exponential growth conditions and a concentration of 2 × 10^9^ bacteria/mL. Bacterial suspensions (25 µL) were exposed to the test product (20 µL) in the absence or presence of metabolic activation (100 µL of S9 mix) for 30 min. Then they were poured into 6-well plates, containing 0.5 mL per well of minimal agar containing biotin and traces of histidine, to allow a few bacteria divisions. A commercial post-mitochondrial liver fraction from rat (S9) was used at 10% *v*/*v* (S9 mix), as the metabolic activation system.

The assay included three technical replicates. In addition, a negative control was used for each strain with and without metabolic activation, which was 20 μL/plate of the solvent used, and a positive control, also per strain and per metabolic condition. The positive controls used were: (a) without metabolic activation (0%S9), 4-nitro-o-phenylenediamine (NPD) 10 µg/well for TA97a and 2 µg/well for TA98; (b) with metabolic activation (10%), 2-aminoanthracene (AA) 2 µg/well for TA97a, and 2-aminofluorene (AF) 10 µg/well for strain TA98. All controls were also in triplicate.

### 2.7. Statistical Analysis

The statistical analysis was performed using STATA/MP 14.1 (StataCorp, College Station, TX, USA). Tables show mean values and standard deviation of the values obtained for the three batches analyzed. Results of TBA and PV were analyzed using a one-way ANOVA to compare oils and to compare treatments independently. For the fatty acids and volatile compounds, in each oil, one-way ANOVA was performed to compare the treatments. In all cases, a post hoc Bonferroni test multiple comparisons was used to assess statistically significant differences among samples. A significance value of *p* ≤ 0.05 was applied for all evaluations.

## 3. Results

### 3.1. Fatty Acid Profile

The fatty acid profile composition of oils provides information on processing conditions and could be applied for quality control purposes, to identify the purity or the mixture of oils [41].

Table 1 shows the summary of the fatty acid profile for the three analyzed oils. Regarding unheated samples (T0), great differences can be observed among the three oils; coconut oil is the most saturated (89.9% SFA), rapeseed oil is the most monounsaturated (65.3% MUFA) and grape seed oil is the most polyunsaturated one (63.8% PUFA). These data agreed with the nature of these oils. Martin et al. showed the composition of different oils, finding values of 91.1% SFA for coconut oil, 72.8% MUFA for rapeseed oil and 74.3% PUFA for grape seed oil [14]. Although certain variability within the same type of oils can be noticed in the fatty acid profiles of oils depending on the variety analyzed, the geographical location and the cultivar conditions, among other factors, the obtained profiles indicate that the samples could be considered as standard for these types of oils.

Regarding healthy aspects, it has to be noted that the presence of trans fatty acid fraction was very low in the three oils (0.01–0.43%). Concerning the unsaturated/saturated and ω-6/ω-3 ratios, relevant differences among the oils were observed. Rapeseed oil showed the best ratio for PUFA + MUFA/SFA ratio, reaching values of 14, and also the best values for ω-6/ω-3 ratio (approximately 2). This oil is poor in ω-3 fatty acids. However, in this case, its higher amount of oleic acid in detriment of linoleic acid, leads to a good equilibrium between ω-6 and ω-3 amounts. Marventano et al. in a review of evidence in human studies of ω-3 and ω-6 PUFA intake on cardiovascular disease, cancer and depressive disorders, concluded that ω-3 PUFAs have been proved to be beneficial, but the role of ω-6 PUFAs needs to be better assessed. These authors pointed out that only a limited number of clinical studies considered the ω-3:ω-6 PUFAs ratio, rather than reporting contrasting results [42].

Analyzing the effects of cooking and accelerated oxidation conditions (Schaal at 65 °C during 20 days) on the fatty acid composition in the three oils (Table 1, and Supplementary Material Tables S1–S3 for the detailed profiles), only slight quantitative modifications were observed, although in some cases they were statistically significant. In the case of SFA, coconut oil maintained values of 89.4 and 90.4% with accelerated storage and cooking, respectively, rapeseed oil values were 6.7 and 6.8% and grape seed oil values were 11.9 and 12.0%. They were all very similar values to those shown for raw samples (89.9%, 6.6% and 11.5%, for coconut, rapeseed and grapeseed oil, respectively). In the case of MUFA, coconut oil showed values of 8.3 and 7.9% (for 20 days storage and cooking, respectively), rapeseed oil 66.0 and 66.7% and grape seed oil 24.6 and 24.7%. Again, these values were very similar to the MUFA amounts found in raw samples (8.1% for coconut, 65.3% for rapeseed and 24.1% for grape seed oil). Finally, in the case of PUFA, values were 2.1–1.5% in coconut, 26.9–26.2% in rapeseed and 62.6–62.3% in grape seed in storage and cooking conditions, respectively. This PUFA fraction was the one that showed the greatest modifications as compared to the values found in unheated samples (2.0%, 27.8% and 63.8% PUFAs in raw samples of coconut, rapeseed and grape, respectively). Coconut oil showed the highest PUFA relative decrease with cooking, around 0.5 g (27%). However, as their presence in the oil is low, this loss does not affect in a relevant manner the general profile of the oil. Pazzoti et al. found that PUFA was the only fraction showing significant modifications in coconut oil after accelerated storage during 20 days [23], showing a greater decrease as compared to that found in our study (from 3.18% to 1.95%). Decreases of PUFAs in the case of rapeseed oil were 1–1.6 g (3.5–5.8%) and in the case of grape seed oil 1.2–1.4 g (1.8–2%). The analysis of the fatty acid profile of a sunflower and soybean mix oil (50%) heated for 30 h at 180 °C showed decreases in the unsaturated fraction of 2.5% (approx. 2 g) mainly due to the decrease in the amount of linoleic acid [43].

Indeed, the significant differences found between the raw samples and those cooked or stored at 65 °C were not substantially relevant from a nutritional point of view considering the dietary intake of the different oils and their different fatty acid proportions. However, these differences could explain some of the changes found in the parameters related to the oxidation status.

### 3.2. Oxidation Status

The intensity of the oxidation process during accelerated storage and cooking treatment was followed through the determination of different chemical markers. Table 2 show Peroxide values and TBARs values.

Peroxide values, indicating the intensity of the primary oxidation products formation, were 0 in raw coconut oil and also after the accelerated storage, reaching high values only after cooking (20.35 meq O_2_/kg of product). A notable increase in PV during cooking coconut oil at 170 °C for 120 min has also been reported [44]. These results indicate higher stability of the lowest unsaturated oil during accelerated storage. However, in the case of the more unsaturated oils (rapeseed and grape seed), the behavior was very different, with medium values in raw samples (14.57 and 12.23 meq O_2_/kg of product for rapeseed and grape seed, respectively), which increased with storage time (18.14–21.86 meq O_2_/kg) and decreased for cooking conditions. Additionally, it has to be pointed out that the primary oxidation products would be degraded to secondary products explaining the lowest values of cooked samples found in rapeseed and grape seeds. Kiralan et al. found a PV of 12.2 meq O_2_/kg of product in raw cold press grape seed oils, with very significant increases during storage (60 °C 6 days) reaching a PV of 80 meq O_2_/kg [45]. Dedebas et al. obtained PV of 7.25 meq O_2_/kg for raw grape seed oils, reaching 21.68 meq O_2_/kg after 12 months at 4 °C and 58.24 meq O_2_/kg after 12 days at 35 °C [46]. Maszewska et al., analyzing the oxidative stability of selected edible refined oils (peanut, corn, rice bran, grapeseed, and rapeseed) during storage found a 30% decrease in oxidative stability in all oils after 12 months of storage [19].

The most widely used method for the determination of malondialdehyde, which is considered the most representative marker of the secondary products of lipid oxidation, is the TBARs method [3]. Again, coconut oil did not seem to suffer from lipid oxidation during the 20 days of accelerated storage, as no amounts of MDA were detected at times 0, 10 and 20 days. Only cooked samples showed a certain level of TBARs, which was lower than the values shown in the more unsaturated oils.

Regarding the TBARs results obtained for rapeseed and grape seed oils, both in unheated and samples stored at Schaal oven conditions, there were some results that were difficult to explain. Very low values were found for both oils after 20 days of accelerated storage, being lower than those obtained at 10 days of storage and even when unheated, in the case of rapeseed oil. This fact was also found in refined olive oil [47] and in hemp seed oil [20] and it was explained as a consequence of the formation of yellow chromophores from the aldehydes (which were formed in high amounts during intense oxidation conditions) which do not absorb at 532 nm. Papastergiadis et al. measuring TBARs in different oils (corn, sunflower, colza and olive) after heating for 6 days at 75 °C, concluded that it is a reliable test to measure the MDA formation, and found the highest values for colza oils (11.75 microg/g) [48]. Berasategi et al. found TBARs around 2–3 mg MDA/kg for avocado and olive oils (also monounsaturated oils) heated for 9 h at 180 °C [49]. These results seemed to indicate that the TBARs test, although measuring the amount of MDA adequately, could show other problems depending on the nature of the oil or conditions of the treatment, so the evaluation of the lipid oxidation in food matrices has to be completed with other oxidation parameters.

### 3.3. Volatile Compounds

The amount and type of volatile compounds are also indicators for the degradation suffered by lipid compounds during oxidation processes. As with the rest of the secondary products, the type of volatile compound formed depends on the fatty acid substrate [2,6].

Our study identified a total of 35 compounds including hydrocarbons, aldehydes, ketones, acids, alcohols, furans and terpenes in the different oil samples (Table 3, Table 4 and Table 5 for coconut, rapeseed and grape seed oil, respectively).

Unheated samples showed very few volatile compounds compared with samples subjected to accelerated storage and, especially cooking conditions. Cooking at 180 °C for 90 min increased the total area reported for unheated samples 18-fold (for rapeseed oil), 30-fold (for grape seed oil) and 35-fold (for coconut). However, for accelerated storage conditions, relative increments were lower than for cooking, and no relevant quantitative differences were found between the two times tested (T1 and T2). Particularly, coconut oil increased, with the storage at 65 °C, 3–4 fold the area detected in unheated samples, whereas the two unsaturated oils showed a 10–11 fold increase after 10 or 20 days of storage, respectively.

Looking into the detailed profiles, raw coconut oil showed the lowest number of compounds with just five volatiles identified, 1,2,3-propanotriol triacetate the most predominant (accounting for approximately 50% of total area), followed by terpenes (30% of total area). With cooking, the amount of total volatiles increased significantly, 24 new compounds were detected, highlighting the significant increase in the saturated aldehydes hexanal, octanal, and nonanal, the unsaturated E-2-heptenal, as well as the relevant amount of 2-pentyl furan, all of them markers of oxidation [20]. Thus, aldehydes accounted for 60% of the total area reported in coconut cooked samples. This sharp increment in volatile aldehydes agreed with that reported by Katragadda et al. [26] in this oil when heated above its smoking point (175°), suggesting that this oil should not be used for deep-fat frying. Moreover, this increment in aldehydes is related to the values observed for PV and TBARs, which showed significant changes in cooked samples for this oil. However, results found during accelerated storage pointed out a slight degree of oxidation, with increments of mostly aldehydes, that accounted for 30–45% of the total area in T1 and T2 samples, respectively. It was interesting to note, contrary to what was observed in the other two oils, the high amounts of octanoic and hexanoic acids, which are characterized by their “fatty” flavor. These fatty acids have also been previously reported in virgin coconut oil treated by different non-thermal processing methods [50].

Grape seed oil, and especially rapeseed oil, showed much more appreciable amounts of volatile compounds in unheated samples as compared to coconut oil. In both cases, the aldehydes family was the predominant one, accounting for approximately 65% (grape seed) 75% (rapeseed) of the total area, with hexanal as the major compound, followed by (E,E)-2,4-heptadienal in rapeseed oil and (E)-2-heptenal in grape seed oil. The higher amount of aldehydes in rapeseed oil was in agreement with the highest values found for TBARs in these samples. High values of aldehydes (hexanal and 2-octenal, mainly) were found during accelerated storage of rapeseed oil (16 days at 60 °C) [51]. Additionally, in accelerated storage, Mildner-Szkudlarz et al. found that secondary oxidation products accounted for approximately 60% of total volatile compounds [21]. In our work, total aldehydes showed the highest increase during storage at 60 °C and especially as a consequence of cooking at 180 °C, reaching in this case, values of around 80% of the total area in the case of rapeseed and 90% for grape seed. Consequently, ketones and furans were more abundant in stored samples than in those submitted to cooking. The most abundant volatiles found in cooked rapeseed samples were 2,4-Heptadienal, (E,E) 2,4-heptadienal, nonanal, (E,E) 2,4-decadienal. 2,4-hepadienal has been described as a typical linolenic acid oxidation product, and it was reported as highly abundant in rapeseed oil treated at 160 °C for more than 30 min [52]. However, in the case of grape seed oil, the two decadienal isomers (2,4-decadienal and (E,E)-2,4-decadienal) were by far the most abundant compounds. These two odor-active compounds are associated with fried fat aroma [53] and oil rancidity [54]. They form chromophores that absorb at 390 nm in the TBARs reaction, and not at 532 nm [47], so this method underestimates oxidation detection in those oils that give rise to these compounds. Regarding nonanal, it is related to the decomposition of hydroperoxides formed by the autoxidation of oleic acid [55] and reached the highest value in cooked samples of the highest monounsaturated oil (rapeseed oil).

Jeleń et al. analyzing volatile compounds in refined and cold press rapeseed oils during 10 days of storage at 60 °C found a significant increase in volatiles, especially of aldehydes, that were higher in the case of refined samples [22]. They also found that in refined oils, 2-hexenal was the most abundant compound with higher increases than the rest of the aldehydes and that 2-heptenal was correlated with the worst samples of refined rapeseed oil stored during 10 days.

Terpenes are usually present in fresh oils. Their amounts in unheated oil were lower in rape seed oil than in coconut and grape seed oils. In all cases, they significantly decreased during the storage, but mostly with cooking (even disappearing in the case of rapeseed oil). These results agree with other papers in which this family of compounds was analyzed during different treatments of oils. A significant decrease in terpene content was also noticed in sunflower oil when being subjected to deep-frying [17] and in hempseed oil during 18 days of storage at 60 °C [20].

In relation to some toxic volatiles formed as a consequence of the degradation basically of ω 3 fatty acids, such as EE-2,4-heptadienal, EE-3,5 octadien-2-one and EE-2,4-decadienal, they were scarcely detected in coconut samples, except for the certain amount of EE-2,4-heptadienal in cooked samples. Regarding the rest of the oils, the highest amounts were found after 20 days of storage and cooked rapeseed samples for EE-3,5-octadien-2-one (which was not detected in grape seed samples) and EE-2,4-heptadienal. EE-2,4-decadienal, as previously stated, reached very high amounts in grape seed samples.

### 3.4. Genotoxic Activity

The vegetable oils extracts did not show a mutation capability because the number of revertant colonies do not differ from the respective negative controls (Table 6). The criteria to consider a positive result was to duplicate the number of revertants in TA97a and/or triplicate the number of revertants in TA98. Thus, negative results have been obtained for all the samples. It has to be mentioned that the negative controls, which indicate the number of spontaneous histidine revertant colonies (mutants), were within the expected values; also, the positive controls were within the expected or historical values for each one of the positive compounds with or without metabolic activation. This is a preliminary study but the results are clearly negative. Nevertheless, it would be convenient to extend the study to the five strains recommended by the TG471 [32] and also to perform an in vitro micronucleus test. Thus, the different mutation endpoints could be covered.

## 4. Conclusions

Changes in the composition of coconut, rapeseed and grape seed oils at different processing conditions were assessed both from the nutritional and safety points of view. No great changes in their fatty acid profile occurred during their storage (65 °C/20 days) or when cooked at 180 °C for 90 min. However, many new compounds of different natures and different amounts depending on their fatty acid profiles are formed during these processes. The more saturated oil (coconut oil) gave rise to a volatile profile characterized by saturated aldehydes and acids under intense heat treatment (180 °C/90 min), whereas unsaturated oils led to significant increases in hexanal, and particularly dienals (heptadienals in rapeseed oil and decadienals in grape seed oil). Regarding safety aspects, no genotoxic activity was detected as a consequence of the applied storage and cooking conditions.

## Figures and Tables

**Table 1:** Fatty acid profile (g/100 g fatty acids) and health-related ratios for the three oils: unheated (T0), after 20 days at 65 °C (T2) and after 90 min at 180 °C (T3).

FATTY ACIDS	T0	T2	T3
Coconut oil			
SFA	89.88 ± 0.07 ^a^	89.43 ± 0.12 ^a^	90.42 ± 0.12 ^b^
MUFA	8.07 ± 0.05 ^b^	8.31 ± 0.09 ^b^	7.90 ± 0.11 ^a^
PUFA	2.03 ± 0.02 ^c^	2.10 ± 0.02 ^b^	1.49 ± 0.01 ^a^
ω-3	ND	ND	ND
ω-6	2.03 ± 0.02 ^b^	2.10 ± 0.02 ^b^	1.49 ± 0.01 ^a^
ω-6/ω-3	-	-	-
PUFA/SFA	0.02 ± 0.00 ^c^	0.02 ± 0.00 ^b^	0.01 ± 0.00 ^a^
PUFA + MUFA/SFA	0.11 ± 0.00 ^b^	0.11 ± 0.00 ^b^	0.10 ± 0.00 ^a^
Trans	0.01 ± 0.00 ^a^	0.05 ± 0.01 ^b^	0.09 ± 0.01 ^c^
Rapeseed oil			
SFA	6.59 ± 1.23	6.75 ± 0.02	6.86 ± 0.07
MUFA	65.33 ± 0.99 ^a^	66.03 ± 0.12 ^ab^	66.66 ± 0.70 ^b^
PUFA	27.84 ± 0.32 ^c^	26.87 ± 0.09 ^b^	26.23 ± 0.36 ^a^
ω-3	9.14 ± 0.13 ^c^	8.69 ± 0.07 ^b^	8.28 ± 0.32 ^a^
ω-6	18.59 ± 0.22 ^b^	18.13 ± 0.04 ^a^	18.22 ± 0.23 ^a^
ω-6/ω-3	2.03 ± 0.03 ^a^	2.08 ± 0.01 ^a^	2.27 ± 0.06 ^b^
PUFA/SFA	4.22 ± 0.62	3.97 ± 0.01	3.82 ± 0.03
PUFA + MUFA/SFA	14.13 ± 2.09	13.75 ± 0.04	13.53 ± 0.06
Trans	0.18 ± 0.01 ^a^	0.27 ± 0.01 ^b^	0.26 ± 0.06 ^b^
Grape seed oil			
SFA	11.49 ± 0.12 ^a^	11.87 ± 0.10 ^b^	11.97 ± 0.09 ^b^
MUFA	24.07 ± 0.14 ^a^	24.65 ± 0.00 ^b^	24.74 ± 0.07 ^b^
PUFA	63.77 ± 0.33 ^b^	62.59 ± 0.15 ^a^	62.39 ± 0.20 ^a^
ω-3	0.05 ± 0.07	0.16 ± 0.02	0.15 ± 0.06
ω-6	63.62 ± 0.36 ^b^	62.37 ± 0.19 ^a^	62.16 ± 0.25 ^a^
ω-6/ω-3	1204.98 ± 458.22	378.27 ± 30.81	411.44 ± 550.61
PUFA/SFA	5.54 ± 0.08 ^b^	5.27 ± 0.06 ^a^	5.20 ± 0.06 ^a^
PUFA + MUFA/SFA	7.64 ± 0.10 ^b^	7.34 ± 0.07 ^a^	7.27 ± 0.07 ^b^
Trans	0.43 ± 0.15	0.49 ± 0.04	0.60 ± 0.07

Values of means ± standard deviations (*n* = 12). Values with different letters for each row are statistically different *p* < 0.05 according to the Bonferroni post hoc test. “ND” reflects that fatty acid was not detected in the sample. “-“: not applicable.

**Table 2:** PV values (meq O_2_/kg of product) and TBARs values (mg MDA/kg of product) for the three oils: unheated (T0), after 10 and 20 days at 65 °C (T1 and T2, respectively) and after 90 min at 180 *°*C (T3).

	Oil	T0	T1	T2	T3
	Coconut	0 ± 0 ^aA^	0 ± 0 ^aA^	0 ± 0 ^aA^	20.35 ± 2.16 ^aA^
PV	Rapeseed	14.57 ± 1.02 ^bC^	16.30 ± 4.69 ^bC^	18.14 ± 6.32 ^cC^	3.12 ± 0.32 ^aC^
	Grape seed	12.23 ± 2.19 ^bB^	22.51 ± 3.52 ^cB^	21.86 ± 5.51 ^dB^	2.47 ± 0.31 ^aB^
	Coconut	0 ± 0 ^aA^	0 ± 0 ^aA^	0 ± 0 ^aA^	2.19 ± 0.10 ^bA^
TBARs	Rapeseed	6.37 ± 0.87 ^bC^	5.51 ± 0.72 ^bC^	1.96 ± 0.10 ^aB^	7.57 ± 0.85 ^cC^
	Grape seed	1.11 ± 0.22 ^aB^	3.69 ± 0.67 ^bB^	1.09 ± 0.11 ^aC^	3.92 ± 0.23 ^bB^

Small letters in the rows indicate statistically significant differences (*p* < 0.05) among treatments for the same oil (Bonferroni post hoc test). Different capital letters in the columns (within each parameter) represent statistically significant differences (*p* < 0.05) among the three oils for each treatment (Bonferroni post hoc test). Mean values ± standard deviations.

**Table 3:** Volatile compound content (*) detected for coconut oil: unheated (T0), after 10 and 20 days of treatment at 65 °C (T1 and T2, respectively) and after 90 min at 180 °C (T3).

Volatile	KI	T0	T1	T2	T3
**Hydrocarbons**					
2-E-Octene	809	ND	6691 ± 4688	ND	ND
2-Z-Octene	816	ND	4685 ± 3378	ND	ND
*Total hydrocarbons (Σ)*		*ND*	11,376 ± 8066	*ND*	*ND*
**Aldehydes**					
(E)-2-Pentenal	748	ND	3983 ± 2086 ^b^	ND	1850 ± 530 ^a^
Hexanal	803	2200 ± 749 ^a^	15,749 ± 2794 ^b^	21,685 ± 1020 ^c^	94,823 ± 7218 ^d^
(E)-2-Hexenal	851	ND	1797 ± 803 ^a^	ND	14,301 ± 901 ^b^
Heptanal	901	ND	1800 ± 387 ^a^	2726 ± 894 ^a^	28,217 ± 3317 ^b^
(E)-2-Heptenal	954	ND	5055 ± 3036 ^a^	8054 ± 934 ^a^	150,112 ± 5215 ^b^
(E,E)-2,4-Heptadienal	995	ND	ND	ND	18,217 ± 1604
Octanal	1002	ND	5462 ± 3111 ^a^	5527 ± 2027 ^a^	207,762 ± 23,477 ^b^
2,4-Heptadienal	1009	ND	2225 ± 267 ^a^	2796 ± 278 ^a^	4968 ± 1704 ^b^
Nonanal	1103	ND	1122 ± 395 ^a^	1176 ± 424 ^a^	106,442 ± 15,688 ^b^
*Total aldehydes (Σ)*		2200 ± 749 ^a^	37,196 ± 12,884 ^a^	41,965 ± 5580 ^a^	626,692 ± 59,654 ^b^
**Ketones**					
2-Hexanone	792	ND	ND	ND	13,732 ± 1592
2-Heptanone	890	ND	1833 ± 627 ^a^	2108 ± 146 ^a^	30,275 ± 3422 ^b^
4-Octanone	972	ND	ND	ND	1957 ± 443
1-Octen-3-one	977	ND	ND	ND	2063 ± 391
3-Octanone	986	ND	ND	ND	3547 ± 851
3-Octen-2-one	1038	ND	468 ± 205	ND	ND
2-Nonanone	1090	ND	ND	ND	30,845 ± 9023
3,5-Octadien-2-one	1092	ND	1322 ± 214	ND	ND
*Total ketones (Σ)*		*ND*	3623 ± 1046 ^a^	2108 ± 146 ^a^	82,419 ± 15,722 ^b^
**Acids**					
Butanoic	793	ND	ND	ND	11,464 ± 1457
Pentanoic	904	ND	ND	ND	11,422 ± 2097
Hexanoic	992	2987 ± 1083 ^a^	8430 ± 1157 ^b^	9074 ± 948 ^b^	16,186 ± 2787 ^c^
Heptanoic	1082	ND	ND	ND	5977 ± 2253
Octanoic	1176	ND	11,783 ± 3971 ^a^	9746 ± 2340 ^a^	37,241 ± 12,031 ^b^
Nonanoic	1276	ND	ND	ND	8294 ± 5203
*Total acids (Σ)*		2987 ± 1083 ^a^	18,053 ± 6590 ^b^	18,820 ± 3289 ^b^	90,584 ± 25,828 ^c^
**Alcohols**					
1-Pentanol	762	ND	2255 ± 607 ^a^	2527 ± 199 ^a^	23,617 ± 2435 ^b^
1-Hexanol	870	ND	1748 ± 2052 ^ab^	454 ± 88 ^a^	2870 ± 702 ^b^
1-Heptanol	970	ND	914 ± 633 ^a^	ND	8945 ± 2214 ^b^
1-octen-3-ol	979	ND	1112 ± 404 ^a^	ND	16,833 ± 857 ^b^
1-Octanol	1072	ND	ND	ND	14,544 ± 5097
*Total alcohols (Σ)*		*ND*	6029 ± 2217 ^a^	2981 ± 266 ^a^	66,809 ± 10,615 ^b^
**Other**					
1,2,3-Propanotriol, triacetate	1362	15,242 ± 8033 ^a^	17,477 ± 4521 ^a^	7572 ± 3551 ^a^	62,884 ± 32,025 ^b^
**Furans**					
Furan-2-Pentyl	991	ND	13,307 ± 8451 ^a^	7694 ± 2791 ^a^	103,251 ± 6151 ^b^
*Total furans (Σ)*		*ND*	13,307 ± 8451 ^a^	7694 ± 2791 ^a^	103,251 ± 6151 ^b^
**Terpenes**					
Alpha-pinene	929	2953 ± 228 ^ab^	3883 ± 1772 ^b^	2083 ± 696 ^a^	ND
Delta-3-carene	1006	ND	6640 ± 1319 ^b^	5411 ± 413 ^a^	ND
Limonene	1025	5889 ± 1338 ^b^	6032 ± 1385 ^b^	5249 ± 1006 ^b^	2857 ± 592 ^a^
*Total terpenes (Σ)*		8842 ± 1566	16,555 ± 4476	12,743 ± 2115	2857 ± 592
***Total Σ***		**29,271 ± 11,431 ^a^**	**123,616 ± 40,215 ^b^**	**93,833 ± 16,285 ^b^**	**1,035,496 ± 151,277 ^c^**

(*) Volatile compounds are expressed per area/sample weight (g) × 10^3^. Values of means ± standard deviations. Values with different letters within rows are statistically different *p* < 0.05 according to the Bonferroni post hoc test. KI: Kovats index. ND:not detected.

**Table 4:** Volatile compound content (*) detected for rapeseed oil: unheated (T0), after 10 and 20 days of treatment at 65 °C (T1 and T2, respectively) and after 90 min at 180 °C (T3).

Volatile	KI	T0	T1	T2	T3
**Hydrocarbons**					
2-E-octene	809	ND	ND	1545 ± 145	1888 ± 536
2-Z-octene	816	ND	ND	1228 ± 147	ND
*Total hydrocarbons (Σ)*		*ND*	*ND*	2773 ± 292	1888 ± 536
**Aldehydes**					
(E)-2-Pentenal	748	3975 ± 250 ^d^	34,862 ± 1209 ^c^	33,744 ± 799 ^b^	22,541 ± 723 ^a^
Hexanal	803	32,637 ± 2008 ^a^	46,946 ± 868 ^b^	54,638 ± 2225 ^c^	90,102 ± 3954 ^d^
(E)-2-Hexenal	851	ND	4812 ± 161 ^a^	7289 ± 406 ^b^	11,901 ± 409 ^c^
Heptanal	901	1328 ± 123 ^a^	4654 ± 403 ^b^	5570 ± 469 ^c^	16,596 ± 677 ^d^
(E,E)-2,4-Hexadienal	908	ND	17,550 ± 4010 ^a^	25,314 ± 1190 ^b^	14,631 ± 1445 ^a^
(E)-2-Heptenal	954	2166 ± 318 ^a^	59,008 ± 738 ^b^	56,474 ± 2760 ^b^	109,829 ± 6641 ^c^
(E,E)-2,4-Heptadienal	995	28,023 ± 1536 ^a^	281,746 ± 9605 ^c^	253,474 ± 4111 ^b^	338,112 ± 13,035 ^d^
Octanal	1002	7425 ± 965 ^a^	38,155 ± 3868 ^b^	48,629 ± 4496 ^c^	108,931 ± 8382 ^d^
2,4-Heptadienal	1009	2024 ± 208 ^a^	123,654 ± 4945 ^b^	142,568 ± 4930 ^c^	406,889 ± 12,144 ^d^
(E)-2-Octenal	1056	ND	20,883 ± 713 ^a^	24,107 ± 1092 ^b^	42,758 ± 1391 ^c^
Nonanal	1103	1783 ± 198 ^a^	25,040 ± 905 ^b^	29,285 ± 1501 ^b^	203,362 ± 9729 ^c^
Nonenal	1158	ND	ND	11,815 ± 1128 ^a^	25,786 ± 988 ^b^
(E,E)-2,4-Decadienal	1291	ND	8795 ± 900 ^a^	15,114 ± 1670 ^a^	199,682 ± 39,899 ^b^
*Total aldehydes (Σ)*		79,361 ± 5606 ^a^	666,105 ± 28,325 ^b^	708,021 ± 26,777 ^c^	1,591,120 ± 98,967 ^d^
**Ketones**					
3-Hexen-2-one	840	ND	808 ± 108 ^a^	ND	5386 ± 229 ^b^
2-Heptanone	890	ND	3011 ± 326 ^a^	3759 ± 406 ^b^	4362 ± 423 ^c^
Ethanone,1-(1-cyclohexen-1-l)	935	ND	148,467 ± 38,941	155,965 ± 2756	170,795 ± 9153
1-Octen-3-one	977	ND	1015 ± 103 ^a^	890 ± 122 ^a^	1513 ± 220 ^b^
3-Octanone	986	ND	ND	ND	5658 ± 705
6-Methyl-5-hepten-2-one	988	ND	10,466 ± 357 ^a^	12,181 ± 198 ^b^	ND
3-Octen-2-one	1038	1845 ± 72 ^a^	9629 ± 353 ^c^	12,298 ± 426 ^b^	3318 ± 230 ^d^
(E,E)-3,5-Octadien-2-one	1070	ND	54,005 ± 968 ^c^	49,940 ± 1116 ^b^	5106 ± 644 ^a^
3,5-Octadien-2-one	1092	ND	45,824 ± 1023 ^a^	51,604 ± 1782 ^b^	ND
*Total ketones (Σ)*		1845 ± 72 ^a^	273,225 ± 42,179 ^c^	286,637 ± 6806 ^b^	196,138 ± 7310 ^d^
**Acids**					
Hexanoic	992	2977 ± 149 ^a^	20,149 ± 971 ^c^	27,373 ± 1747 ^d^	5657 ± 421 ^b^
Octanoic	1176	ND	ND	ND	4000 ± 1309
Nonanoic	1276	ND	ND	ND	8768 ± 2728
*Total acids (Σ)*		2977 ± 149 ^a^	20,149 ± 971 ^b^	27,373 ± 1747 ^b^	18,425 ± 4458 ^b^
**Alcohols**					
1-Pentanol	762	2554 ± 123 ^a^	ND	ND	26,434 ± 703 ^b^
1-Hexanol	870	6599 ± 255 ^d^	1628 ± 77 ^b^	1239 ± 111 ^a^	2223 ± 295 ^c^
1-Heptanol	970	ND	ND	ND	11,367 ± 923
1-Octen-3-ol	979	638 ± 78 ^a^	14,992 ± 436 ^c^	13,249 ± 622 ^b^	18,115 ± 1042 ^d^
1-Octanol	1072	ND	ND	ND	17,633 ± 631
*Total alcohols (Σ)*		9791 ± 456 ^a^	16,620 ± 513 ^b^	14,488 ± 733 ^b^	75,772 ± 3594 ^c^
**Furans**					
Furan, 2-Pentyl	991	7891 ± 1559 ^a^	58,918 ± 2481 ^c^	82,661 ± 4614 ^d^	50,624 ± 2529 ^b^
*Total furans (Σ)*		7891 ± 1559 ^a^	58,918 ± 2481 ^c^	82,661 ± 4614 ^d^	50,624 ± 2529 ^b^
**Terpenes**					
Alpha-pinene	929	368 ± 25	ND	ND	ND
Limonene	1025	1897 ± 207	1871 ± 147	1835 ± 572	ND
*Total terpenes (Σ)*		2265 ± 232	1871 ± 147	1835 ± 572	*ND*
***Total Σ***		**104,130 ± 8074 ^a^**	**1,036,888 ± 74,616 ^b^**	**1,123,788 ± 41,541 ^b^**	**1,933,967 ± 22,096 ^c^**

(*) Volatile compounds are expressed per area/sample weight (g) × 10^3^. Values of means ± standard deviations. Values with different letters within rows are statistically different *p* < 0.05 according to the Bonferroni post hoc test. KI: Kovats index. ND:not detected.

**Table 5:** Volatile compound content (*) detected for grape seed oil: unheated (T0), after 10 and 20 days of treatment at 65 °C (T1 and T2, respectively) and after 90 min at 180 °C (T3).

Volatile	KI	T0	T1	T2	T3
**Hydrocarbons**					
1-Octene	793	1328 ± 133 ^a^	ND	ND	2043 ± 346 ^b^
2-E-octene	809	5904 ± 500 ^b^c	4222 ± 432 ^a^	6663 ± 531 ^c^	5456 ± 1070 ^b^
2-Z-octene	816	2495 ± 264 ^b^c	1701 ± 129 ^a^	2827 ± 338 ^c^	2178 ± 514 ^b^
*Total hydrocarbons (Σ)*		9727 ± 897	5923 ± 561	9490 ± 869	9677 ± 1930
**Aldehydes**					
(E)-2-Pentenal	748	ND	2579 ± 153	3011 ± 622	ND
Hexanal	803	35,088 ± 5643 ^a^	66,528 ± 4110 ^b^	66,147 ± 3541 ^b^	137,213 ± 12,039 ^c^
(E)-2-Hexenal	851	747 ± 103 ^a^	23,070 ± 1323 ^b^	21,868 ± 1717 ^b^	30,594 ± 3326 ^c^
Heptanal	901	1432 ± 176 ^a^	5832 ± 1322 ^b^	5033 ± 504 ^b^	11,212 ± 1614 ^c^
(E)-2-Heptenal	954	8510 ± 596 ^a^	208,336 ± 8588 ^b^	199,345 ± 11,880 ^b^	270,474 ± 35,611 ^c^
(E,E)-2,4-Heptadienal	995	1644 ± 193 ^a^	24,758 ± 1671 ^b^	28,718 ± 3293 ^b^	46,073 ± 5513 ^c^
Octanal	1002	2698 ± 321 ^a^	31,674 ± 2623 ^b^	38,800 ± 3559 ^b^	79,232 ± 12,472 ^c^
2,4-Heptadienal	1009	ND	6097 ± 487 ^a^	7755 ± 524 ^b^	17,729 ± 1459 ^c^
(E)-2-Octenal	1056	1484 ± 142 ^a^	57,966 ± 9692 ^b^	79,807 ± 8360 ^c^	86,214 ± 12,884 ^c^
Nonanal	1103	693 ± 65 ^a^	3789 ± 553 ^a^	2914 ± 229 ^a^	56,156 ± 6510 ^b^
(E,E)-2,4-Octadienal	1107	ND	860 ± 68 ^a^	ND	2246 ± 300 ^b^
Nonenal	1158	ND	7134 ± 365 ^a^	13,697 ± 691 ^b^	16,610 ± 1021 ^c^
2,4-Nonadienal	1212	ND	4120 ± 244 ^a^	4784 ± 367 ^a^	13,199 ± 2519 ^b^
(E,E)-2,4-Decadienal	1291	ND	13,561 ± 964 ^a^	20,207 ± 1463 ^a^	672,012 ± 168,979 ^b^
2,4-Decadienal	1317	ND	33,428 ± 2592 ^a^	56,523 ± 4908 ^a^	727,265 ± 142,179 ^b^
*Total aldehydes (Σ)*		52,296 ± 7239 ^a^	489,732 ± 34,255 ^b^	548,612 ± 22,893 ^b^	2,166,229 ± 236,907 ^c^
**Ketones**					
2-Heptanone	890	ND	7908 ± 804 ^b^	9069 ± 896 ^a^	5442 ± 1112 ^c^
1-Octen-3-one	977	ND	2898 ± 92 ^a^	2337 ± 293 ^b^	2312 ± 566 ^a^
3-Octanone	986	ND	2245 ± 123 ^c^	2936 ± 258 ^b^	1522 ± 290 ^a^
3-Octen-2-one	1038	ND	26,868 ± 1438 ^b^	32,210 ± 6306 ^c^	5951 ± 951 ^a^
(E,E)-3,5-Octadien-2-one	1070	ND	1332 ± 137	ND	ND
3,5-Octadien-2-one	1092	ND	1521 ± 243	ND	ND
*Total ketones (Σ)*		ND	42,772 ± 2837 ^b^	46,552 ± 2077 ^b^	15,227 ± 2919 ^a^
**Acids**					
Hexanoic	992	3202 ± 589 ^a^	72,354 ± 10,221 ^b^	89,608 ± 8801 ^c^	8441 ± 2634 ^a^
*Total acids (Σ)*		3202 ± 589 ^a^	72,354 ± 10,221 ^b^	89,608 ± 8801 ^c^	8441 ± 2634 ^a^
**Alcohols**					
1-Pentanol	762	1243 ± 194 ^a^	3902 ± 541 ^a^	3868 ± 405 ^a^	61,807 ± 5740 ^b^
1-Hexanol	870	ND	1796 ± 109 ^a^	2188 ± 239 ^b^	1809 ± 309 ^a^
1-Heptanol	970	ND	ND	ND	5429 ± 892
1-Octen-3-ol	979	997 ± 232 ^a^	53,096 ± 2482 ^b^	48,707 ± 2419 ^b^	51,296 ± 5752 ^b^
2-Octen-1-ol	1068	ND	ND	ND	5203 ± 505
1-Octanol	1072	ND	ND	ND	8359 ± 901
*Total alcohols (Σ)*		2240 ± 426 ^a^	58,794 ± 3132 ^b^	54,763 ± 3063 ^b^	133,903 ± 14,099 ^c^
**Furans**					
Furan, 2-Pentyl	991	7101 ± 506 ^a^	100,415 ± 13,052 ^c^	163,693 ± 17,544 ^d^	57,458 ± 9410 ^b^
*Total furans (Σ)*		7101 ± 506 ^a^	100,415 ± 13,052 ^c^	163,693 ± 17,544 ^d^	57,458 ± 9410 ^b^
**Terpenes**					
Alpha-pinene	929	274 ± 202	ND	ND	ND
Para-cymene	1022	3002 ± 251	ND	ND	ND
Limonene	1025	3955 ± 3676 ^a^	13,349 ± 166 ^b^	ND	907 ± 423 ^a^
*Total terpenes (Σ)*		7231 ± 4129 ^b^	13,349 ± 166 ^c^	*ND*	907 ± 423 ^a^
***Total Σ***		**81,797 ± 13,786 ^a^**	**783,339 ± 64,224 ^b^**	**912,722 ± 51,226 ^b^**	**2,391,856 ± 217,406 ^c^**

(*) Volatile compounds are expressed per area/sample weight (g) × 10^3^. Values of means ± standard deviations. Values with different letters within rows are statistically different *p* < 0.05 according to the Bonferroni post hoc test. KI: Kovats index. ND:not detected.

**Table 6:** TA97a and TA98 revertant colonies detected after exposure to the three oils (coconut, rapeseed and grape seed) both without (0%) S9) and with metabolic activation (10% S9). Mean ± SD of three replicates is presented. Each of the oils were tested without any treatment (T0), after 10 and 20 days of treatment at 65 °C (T1 and T2, respectively) and after 90 min at 180 °C (T3). Positive controls (C+) were: (a) without metabolic activation (0%S9), 4-nitro-o-phenylenediamine (NPD) 10 µg/well for TA97a and 2 µg/well for TA98; (b) with metabolic activation (10% S9), 2-aminoanthracene (AA) 2 µg/well for TA97a, and 2-aminofluorene (AF) 10 µg/well for strain TA98.

	TA97a		TA98	
	0%S9	10%S9	0%S9	10%S9
C−	36 ± 12	54 ± 5	8 ± 1	12 ± 1
C+	315 ± 32	421 ± 94	317 ± 43	877 ± 82
Coconut oil				
T0	36 ± 5	73 ± 15	10.2	13 ± 5
T1	41 ± 11	57 ± 6	7 ± 1	8 ± 1
T2	33 ± 2	56 ± 3	10 ± 2	11 ± 2
T3	45 ± 3	73 ± 4	10 ± 2	10 ± 3
Rapeseed oil				
T0	35 ± 3	73 ± 2	12 ± 2	13 ± 4
T1	61 ± 9	76 ± 9	10 ± 4	8 ± 3
T2	42 ± 1	70 ± 6	7 ± 5	15 ± 7
T3	52 ± 10	73 ± 3	10 ± 1	11 ± 2
Grape seed oil				
T0	45 ± 4	73 ± 5	15 ± 3	12 ± 1
T1	47 ± 6	75 ± 11	13 ± 2	11 ± 2
T2	48 ± 7	72 ± 11	11 ± 3	10 ± 3
T3	39 ± 7	67 ± 9	11 ± 4	16 ± 11

## Data Availability

Data is contained within the article or Supplementary Materials.

## Supplementary Materials

The following supporting information can be downloaded at: https://www.mdpi.com/article/10.3390/foods12112186/s1, Table S1: Fatty acid profile (g/100 g fatty acids) and health-related ratios for coconut oil: unheated (T0), after 20 days at 65 °C (T2) and after 90 min at 180 °C (T3). Table S2: Fatty acid profile (g/100 g fatty acids) and health-related ratios for rapeseed oil: unheated (T0), after 20 days at 65 °C (T2) and after 90 min at 180 °C (T3). Table S3: Fatty acid profile (g/100 g fatty acids) and health-related ratios for grape seed oil: unheated (T0), after 20 days at 65 °C (T2) and after 90 min at 180 °C (T3).
