# Supplementary material for: Oxidative Stability and Genotoxic Activity of Vegetable Oils Subjected to Accelerated Oxidation and Cooking Conditions
Source: Foods. 2023 May 29;12(11):2186. doi: 10.3390/foods12112186 (PMC10252270; doi:10.3390/foods12112186)
Supplement: Supplementary file 1 [file foods-12-02186-s001.zip › foods-2425464-supplementary.pdf]

Supplementary material Ansorena et al. – *Foods*- Oxidative stability and genotoxic activity of vegetable oils subjected to accelerated oxidation and cooking conditions

Table S1: Fatty acid profile (g/100g fatty acids) and health-related ratios for coconut oil: unheated (T0), after 20 days at 65°C (T2) and after 90min at 180°C (T3).

Fatty acids	T0	T2	T3
Caprilic C8:0	5.25±0.12b	5.39±0.12b	5.07±0.03a
Capric C10:0	5.04±0.07b	5.07±0.03b	4.93±0.04a
Lauric C12:0	44.52±0.14	44.29±0.10	44.48±0.16
Myristic C14:0	20.61±0.10c	20.41±0.04b	21.06±0.02a
Palmitic C16:0	11.03±0.12a	10.93±0.08a	11.37±0.06b
t-Palmitoleic C16:1Δ9t	ND	ND	ND
Palmitoleic C16:1	ND	0.04±0.00b	0.03±0.00a
Stearic C18:0	3.34±0.05a	3.32±0.03a	3.48±0.02b
ΣTrans isomers C18:1	0.01±0.01a	0.05±0.01b	0.09±0.01c
Oleic C18:1	7.87±0.03b	7.99±0.09b	7.67±0.10a
c-Vaccenic C18:1	0.18±0.01b	0.18±0.01b	0.16±0.00a
t-Linoleic C18:2Δ9t. 12t	ND	ND	ND
c-t Linoleic C18:2Δ9c. 12t	ND	ND	ND
t-c Linoleic C18:2Δ9t. 12c	ND	ND	ND
Linoleic C18:2Δ9c. 12c (ω-6)	2.03±0.01b	2.07±0.02c	1.48±0.01a
Arachidic C20:0	0.08±0.01	0.10±0.01	0.10±0.01
γ- Linolenic C18:3 (ω-6)	ND	ND	ND
Eicosenoic C20:1	0.03±0.00ab	0.09±0.01b	0.03±0.01a
α- Linolenic C18:3 (ω-3)	ND	ND	ND
Eicosadienoic C20:2 (ω-6)	ND	ND	ND
Behenic C22:0	ND	ND	ND
t-Brassidic C20:1Δ13t	ND	ND	ND
Erucic C22:1	ND	ND	ND
Eicosatrienoic C20:3 (ω-6)	ND	ND	ND
Arachidonic C20:4 (ω-6)	ND	ND	ND
Lignoceric C24:0	ND	ND	ND
Eicosapentaenoic C20:5 (ω-3)	ND	ND	ND
Nervonic C24:1	ND	ND	ND
Docosatrienoic C22:3	ND	ND	ND
Docosapentaenoic C22:5 (ω-6)	ND	ND	ND
Docosapentaenoic C22:5 (ω-3)	ND	ND	ND
Docosahexaenoic C22:6 (ω-3)	ND	ND	ND

Values of means ± standard deviations (n=12). Values with different letters for each row are statistically different according to the Bonferroni post hoc test. "ND" reflects that fatty acid was not detected in the sample. “-“: not applicable

Table S2: Fatty acid profile (g/100g fatty acids) and health-related ratios for rapeseed oil: unheated (T0), after 20 days at 65°C (T2) and after 90min at 180°C (T3).

Fatty acids	T0	T2	T3
Caprilic C8:0	ND	ND	ND
Capric C10:0	ND	ND	ND
Lauric C12:0	0.03±0.01	0.04±0.02	0.01±0.00
Myristic C14:0	0.03±0.01a	0.05±0.01b	0.04±0.00b
Palmitic C16:0	4.25±0.07a	4.37±0.01b	4.44±0.04b
t-Palmitoleic C16:1Δ9t	ND	ND	ND
Palmitoleic C16:1	0.16±0.00	0.16±0.04	0.13±0.03
Stearic C18:0	1.56±0.03a	1.62±0.00b	1.65±0.02b
ΣTrans isomers C18:1	0.09±0.01a	0.18±0.01b	0.17±0.06b
Oleic C18:1	60.88±0.93a	61.73±0.14b	61.95±0.64b
c-Vaccenic C18:1	3.27±0.04a	3.23±0.01a	3.43±0.03b
t-Linoleic C18:2Δ9t. 12t	ND	ND	ND
c-t Linoleic C18:2Δ9c. 12t	ND	ND	ND
t-c Linoleic C18:2Δ9t. 12c	ND	ND	ND
Linoleic C18:2Δ9c. 12c (ω-6)	18.53±0.21b	18.07±0.044a	18.10±0.23a
Arachidic C20:0	0.52±0.04	0.48±0.06	0.49±0.011
γ- Linolenic C18:3 (ω-6)	ND	ND	ND
Eicosenoic C20:1	0.93±0.02a	0.96±0.00b	0.97±0.01b
α- Linolenic C18:3 (ω-3)	9.21±0.13c	8.62±0.07b	8.01±0.31a
Eicosadienoic C20:2 (ω-6)	0.09±0.01	0.12±0.05	0.12±0.05
Behenic C22:0	0.13±0.01a	0.14±0.00a	0.18±0.01b
t-Brassidic C20:1Δ13t	0.09±0.01	0.09±0.01	0.09±0.01
Erucic C22:1	ND	ND	ND
Eicosatrienoic C20:3 (ω-6)	ND	ND	ND
Arachidonic C20:4 (ω-6)	ND	ND	ND
Lignoceric C24:0	ND	ND	ND
Eicosapentaenoic C20:5 (ω-3)	ND	ND	ND
Nervonic C24:1	ND	ND	ND
Docosatrienoic C22:3	ND	ND	ND
Docosapentaenoic C22:5 (ω-6)	ND	ND	ND
Docosapentaenoic C22:5 (ω-3)	ND	ND	ND
Docosahexaenoic C22:6 (ω-3)	ND	ND	ND

Values of means ± standard deviations (n=12). Values with different letters for each row are statistically different according to the Bonferroni post hoc test. "ND" reflects that fatty acid was not detected in the sample. "-": not applicable

Table S3: Fatty acid profile (g/100g fatty acids) and health-related ratios for grape seed oil: unheated (T0), after 20 days at 65°C (T2) and after 90min at 180°C (T3).

Fatty acids	T0	T2	T3
Caprilic C8:0	0.07±0.07	0.13±0.07	0.07±0.00
Capric C10:0	0.00±7.82b	0.00±0.00a	ND
Lauric C12:0	0.02±0.01b	0.02±0.00b	0.01±0.00a
Myristic C14:0	0.05±0.01	0.05±0.00	0.05±0.00
Palmitic C16:0	7.10±0.09a	7.14±0.03a	7.21±0.07b
t-Palmitoleic C16:1Δ9t	ND	ND	ND
Palmitoleic C16:1	0.12±0.01	0.10±0.01	0.10±0.01
Stearic C18:0	4.01±0.07a	4.16±0.03b	4.19±0.05b
ΣTrans isomers C18:1	0.27±0.07	0.21±0.04	0.27±0.08
Oleic C18:1	22.98±0.15a	23.61±0.01b	23.67±0.06b
c-Vaccenic C18:1	0.80±0.01a	0.81±0.00b	0.81±0.00b
t-Linoleic C18:2Δ9t. 12t	ND	ND	ND
c-t Linoleic C18:2Δ9c. 12t	0.07±0.01a	0.16±0.01b	0.18±0.011b
t-c Linoleic C18:2Δ9t. 12c	0.09±0.02	0.12±0.01	0.15±0.03
Linoleic C18:2Δ9c. 12c (ω-6)	63.57±0.36b	62.44±0.18a	62.43±0.24a
Arachidic C20:0	0.14±0.08	0.16±0.02	0.15±0.00
γ- Linolenic C18:3 (ω-6)	0.03±0.00b	0.03±0.00a	0.03±0.00a
Eicosenoic C20:1	0.12±0.01	0.11±0.00	0.12±0.00
α- Linolenic C18:3 (ω-3)	0.05±0.02	0.16±0.02	0.15±0.06
Eicosadienoic C20:2 (ω-6)	0.05±0.03	0.03±0.02	0.05±0.03
Behenic C22:0	0.10±0.08a	0.18±0.01b	0.12±0.09ab
t-Brassidic C20:1Δ13t	ND	ND	ND
Erucic C22:1	ND	ND	ND
Eicosatrienoic C20:3 (ω-6)	ND	ND	ND
Arachidonic C20:4 (ω-6)	ND	ND	ND
Lignoceric C24:0	ND	ND	ND
Eicosapentaenoic C20:5 (ω-3)	ND	ND	ND
Nervonic C24:1	ND	ND	ND
Docosatrienoic C22:3	ND	ND	ND
Docosapentaenoic C22:5 (ω-6)	ND	ND	ND
Docosapentaenoic C22:5 (ω-3)	ND	ND	ND
Docosahexaenoic C22:6 (ω-3)	ND	ND	ND

Values of means ± standard deviations (n=12). Values with different letters for each row are statistically different according to the Bonferroni post hoc test. "ND" reflects that fatty acid was not detected in the sample. "-": not applicable
